# Reliability of glenoid measurements performed using Multiplanar Reconstruction (MPR) of Magnetic Resonance (MRI) in patients with shoulder instability

**DOI:** 10.1007/s00264-024-06226-0

**Published:** 2024-06-04

**Authors:** Jan Nizinski, Agata Kaczmarek, Bartosz Antonik, Sebastian Rauhut, Piotr Tuczynski, Filip Jakubowski, Julian Slawski, Jakub Stefaniak, Przemyslaw Lubiatowski

**Affiliations:** 1 Rehasport Clinic, Poznan, Poland; 2 Department of Traumatology, Orthopaedics and Hand Surgery, University of Medical Sciences in Poznan, Poznan, Poland

**Keywords:** Glenoid, Shoulder instability, Bone defect, Magnetic resonance, Bone deficiency, Dislocation

## Abstract

**Purpose:**

Measurement of glenoid bone loss in the shoulder instability can be assessed by CT or MRI multiplanar imaging and is crucial for pre-operative planning. The aim of this study is to determine the intra and interobserver reliability of glenoid deficiency measurement using MRI multiplanar reconstruction with 2D assessment in the sagittal plane (MPR MRI).

**Methods:**

We reviewed MRI images of 80 patients with anterior shoulder instability with Osirix software using MPR. Six observers with basic experience measured the glenoid, erosion edge length, and bone loss twice, with at least one-week interval between measurements. We calculated reliability and repeatability using the intra-class correlation coefficient (ICC) and minimal detectable change with 95% confidence (MDC95%).

**Results:**

Intra and Inter-observer ICC and MDC95% for glenoid width and height were excellent (ICC 0,89-0,96). For erosion edge length and area of the glenoid were acceptable/good (ICC 0,61-0,89). Bone loss and Pico Index were associated with acceptable/good ICC (0,63 -0,86)) but poor MDC95% (45 - 84 %). Intra-observer reliability improved with time, while inter-observer remained unchanged.

**Conclusion:**

The MPR MRI measurement of the anterior glenoid lesion is very good tool for linear parameters. This method is not valid for Pico index measurement, as the area of bone loss is variable. The pace of learning is individual, therefore complex calculations based on MPR MRI are not resistant to low experience as opposed to true 3D CT

## Introduction

Shoulder instability is a quite frequent medical condition which affects mostly young and active men. The overall incidence rate is estimated to be around 23.9 per 100 000 person-years [1]. One of the treatment challenges is a very high recurrence rate, estimated to be between 66% to 94% among patients under the age of twenty years [2–5]. As a result of the dislocation, especially recurrent, the bony structures of the joint may be damaged. This includes glenoid bone loss, Hill-Sachs lesions, fractures or any other joint abnormalities. Surgical treatment is commonly indicated. Most of the procedures fall into the category of earth soft tissue repair (arthroscopic Bankart) or osseous procedures (Latarjet, bone block). Bony defects are the main factor in decision-making for a particular procedure to avoid surgical failure. Therefore, it is so important to evaluate the bony defects radiologically. Bone defects in the shoulder joint can be measured using various imaging techniques such as X-ray, computer tomography (CT) and magnetic resonance imaging (MRI). While X-ray is definitely the first choice it has low value for calculations, CT and MRI both can accurately detect glenoid bone loss [6]. So far CT and both 2D and true 3D reconstruction have been a gold standard of bone defects evaluation. Moreover, true 3D showed significantly better accuracy than 2D and also proved to be resistant to less experienced evaluators. Recent technological advancements allow obtaining MRI images, which facilitate 3D reconstruction. In the literature, there is good evidence that 3D CT is more accurate than 2D CT for glenoid and humeral head measurements [7]. Other studies show that 3D MRI is comparable to 3D CT and thus should be taken under consideration as a good, radiation-free, diagnostic technique for measuring glenoid bone loss [8].

MRI is a fairly accessible imaging technique that, unlike X-rays and CT scans, does not expose the patient to X-rays. Additionally, it allows accurate visualization of cartilage and soft tissues, such as muscles, tendons, and ligaments [9]. The choice of MRI scanner for bone examination should be based on the strength of its magnetic field, coil, and pulse sequence. The signal-to-noise ratio (SNR) must be at least 10 to visualize trabecular microarchitecture adequately. MRI with higher magnetic induction units is best suited for this purpose [10].

Despite the increasing use of MRI, there are still few results showing the reliability of both simple and more advanced measurements of glenoid bone loss. Therefore, the primary aim of this study was to determine the reliability of glenoid measurements performed using Multiplanar Reconstruction (MPR) of Magnetic Resonance Imaging (MRI) in patients with shoulder instability. We have hypothesized that MPR MRI is a reliable tool for glenoid measurements. The secondary aim was to assess the impact of learning and experience on such evaluations.

## Materials and methods

This is an observational study. The Poznan University of Medical Science Research Ethics Committee has confirmed that no ethical approval is required. The study included 80 consecutive patients diagnosed with traumatic anterior shoulder instability (63 men and 17 women) with a mean age of 30,2, +/- 15 who underwent an MRI arthrography scan (aMR) in our institution. The MRI examination was performed using a Siemens MAGNETOM Spectra 3T (3-Tesla) instrument. Volumetric interpolated breath-hold examination (VIBE) sequence was used for the evaluation, due to its high internal contrast distribution. VIBE is useful for evaluating the bony structures of the shoulder due to their close proximity to soft tissues. VIBE uses fast 3D gradient-echo sequences to produce T1 images. High-quality multiplanar and 3D reconstruction images are obtained using improved Z-axis resolution. In VIBE sequences, soft tissues have a similar signal to fibrous tissue and mineralizing callus. Intra-cortical bone pathology is clearly visible due to relatively high internal contrast distribution. Therefore, using different MRI sequences during one examination, we can visualize both soft tissue, bone, and cartilage damage. The patient is not exposed to X-rays and does not have to make two appointments for imaging tests [11]. Average acquisition time for shoulder was about 30 minutes. VIBE sequence alone takes around three to four minutes. No discomfort has been noted and non of the images had singes of motion-related artefacts.

## Multiplanar reconstruction

Based on the MRI results, the research group measured the glenoid fossa of the shoulder joint using MPR MRI reconstruction using OsiriX MD (Pixmeo) v.6.5 64-bit software based on echo gradient sequence. Before measuring each glenoid scan was set in the transverse, coronal and sagittal plane. The study was approved by an internal review board. MPR is in fact a two-dimensional assessment allowing for precise adjustment of the evaluation plane (sagittal).

**Fig. 1:**
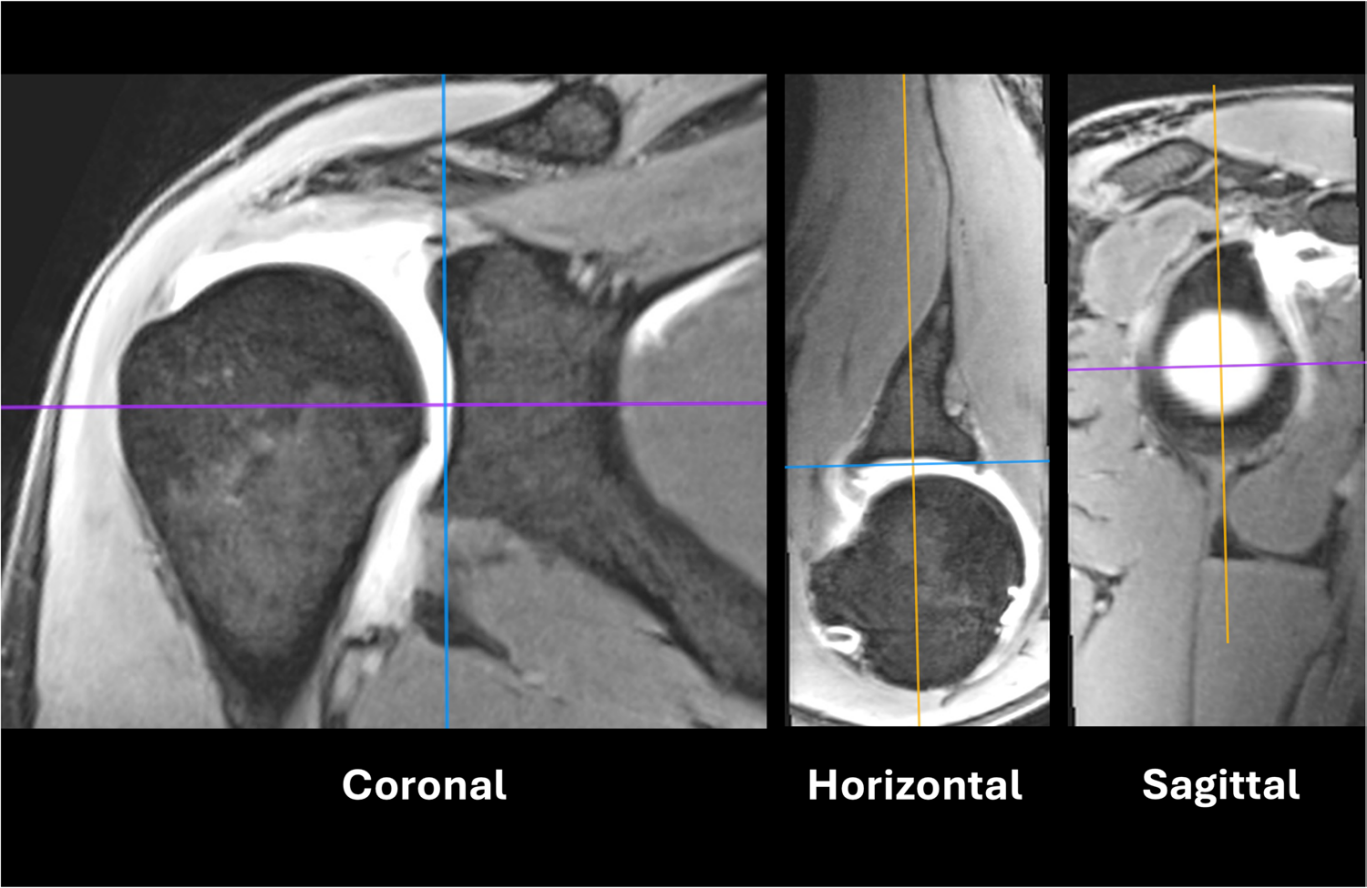
Multiplannar reconstruction based on VIBE sequence of MRI

**Fig. 2:**
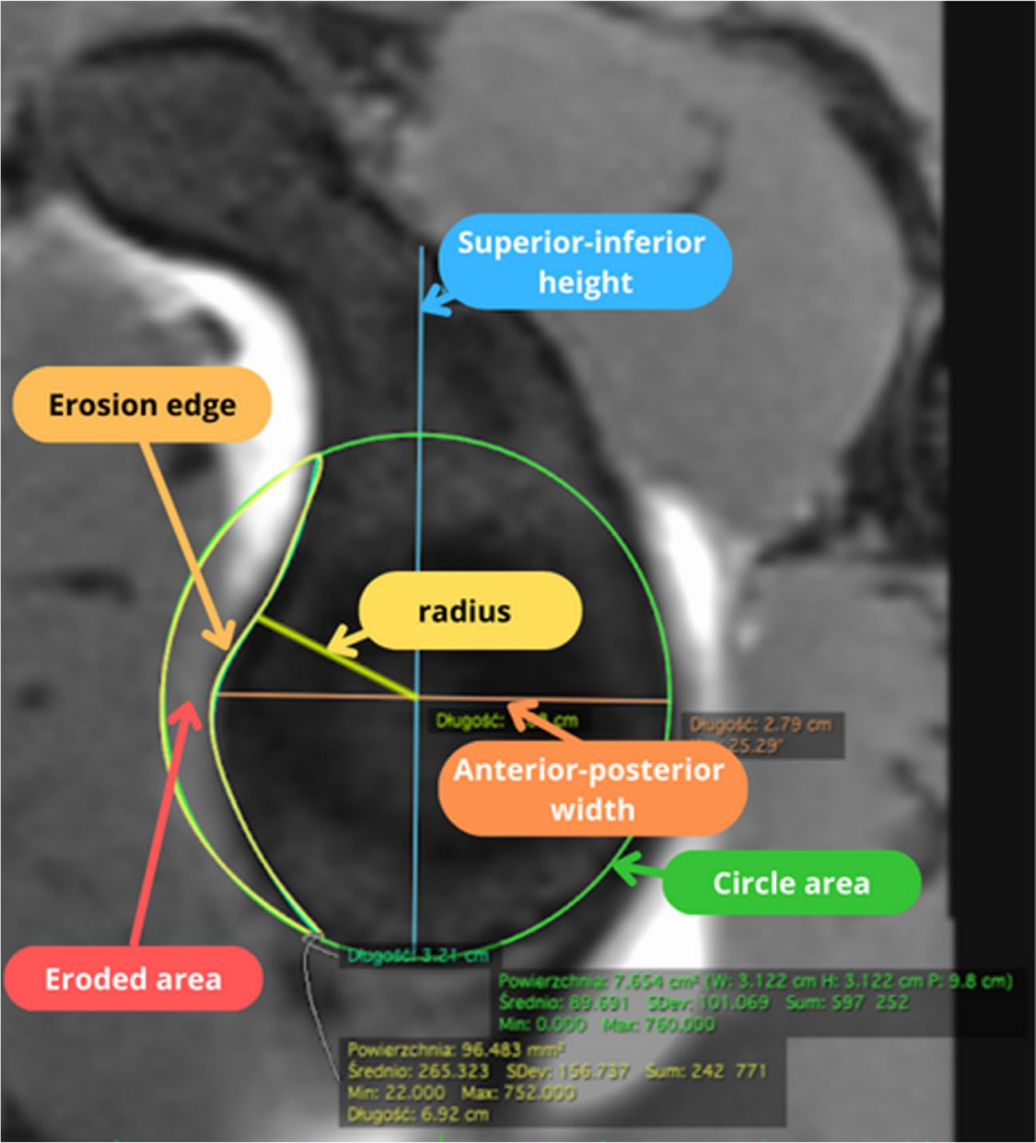
Sample measurement of glenoid parameters on MRI image - vibe sequence

For optimal evaluation and centering of the sagittal plane of the glenoid, the horizontal and coronal planes were positioned in a specific manner. Two lines at right angles were used. One had to pass through the upper and lower edges of the glenoid (in the coronal plane) or the anterior and posterior edges of the glenoid (horizontal plane). The second line marked the centre of the glenoid. Measurements are taken in the sagittal plane, so that the entire glenoid is visible, together with a small fragment of the humeral head or contrast in the center (the glenoid is concave), as shown in Fig. 1. This allowed for repeatable and consistent orientation of the MRI images. From the MRI image oriented in the sagittal plane, seven specific parameters of the glenoid were measured including both simple dimensions and more complex area measurements (Table 1) (Fig. 2). Every MRI image was evaluated twice by a researcher with at least one-week interval between measurements.

**Table 1:** Measured parameters of glenoid

Measurement	Description	Abbreviation	References
Superior-inferior height	The longest distance between the most anterior	SIh	Griffith et al. [12].
Anterior-posterior width	The longest distance between the most anterior and the most posterior part of the glenoid	APw	Gerber and Nyffeler, Griffith et al. [12, 13]
Erosion edge	Length of the glenoid rim bone loss	EE	Gerber and Nyffeler, Griffith et al. [12, 13]
Radius	The radius of the circle area	R	Barchilon et al. [14]
Circle area	Area of the best fitted circle in the inferior glenoid rim	CA	Sugaya et al., Magarelli et al. [15, 16]
Eroded area	Area of the missing part of circle area	EA	Sugaya et al., Magarelli et al. [15, 16]
Pico’s method	The ratio of the EA to the CA multiplied by 100%,	EA ÷CA× 100%	Baudi et al. [17]
Gerber’s Index	The ratio of the EE to the diameter of the inscribed circle multiplied by 100%,	EE ÷diameter× 100%	Gerber and Nyffeler [13]

### Reliability assessment

Each MRI scan was evaluated by each of six independent researchers twice with a minimum one-week interval between each measurement for both inter- and interobserver reliability. The researchers had completed at least three years of medical school and had no previous experience in measuring musculoskeletal parameters on imaging. Each researcher was trained in the basics and techniques of measurement by an experienced shoulder surgeon with major experience in the assessment of glenoids using MRI. In addition, after evaluating the first 20 patients twice, the specialist rated the correctness of the measurement methodology.

For statistical analysis, SPSS Statistics was used. In the statistical analysis, Intraclass Correlation Coefficient (ICC) was calculated. Based on the 95% confident interval of the ICC estimate, values less than 0.5, between 0.5 and 0.75, between 0.75 and 0.9, and greater than 0.90 are indicative of poor, moderate, good, and excellent reliability, respectively [18]. Additionally, minimal detectable change with 95% confidence (MDC95%) was calculated to assess measurement reliability.

The impact of learning was assessed by comparing intra-and interobserver reliability between 30 MRI images that were measured at the beginning of the study and 30 MRI images that were assessed at the end.

## Results

### Intra-observer reliability

Results showed that ICC for superior-inferior height and anterior-posterior width are excellent. MDC95(%) for these parameters was excellent or good (almost excellent) (Table 2). The ICC for the remaining parameters were good. MDC95% was good for Erosion edge, Radius, Circle Area and Gerber’s index. On the other hand, MDC95 was not acceptable in the Eroded area and Pico Index.

**Table 2:** Results of intra-observer and inter-observer reliability. (ICC intraclass correlation coefficient, MDC95 minimal detectable change with 95% confidence). Intra-observer results based on 480 measurements (6 researchers and 80 patients). Inter – observer results based on 160 measurements (80 patients, measured 2 times by each observer)

Parameter	Intra-observer reliability			Inter-observer reliability		
	ICC	95% CI	MDC95 (%)	ICC	95% CI	MDC95 (%)
Superior-inferior height (cm)	0,9106	0,8931 - 0,9253	0,35 (8,08%)	0,9180	0,8778 - 0,9435	0,32 (7,37%)
Anterior-posterior width (cm)	0,9178	0,9017 - 0,9313	0,27 (10,31%)	0,9624	0,9525 - 0,9707	0,18 (6,82%)
Erosion edge (cm)	0,8198	0,7840 - 0,8496	0,85 (25,59%)	0,6159	0,4667 - 0,7224	1,08 (32,40%)
Radius (cm)	0,8474	0,8174 - 0,8725	0,43 (13,45%)	0,8580	0,7862 - 0,9030	0,38 (11,53%)
Circle area (cm2)	0,8948	0,8738 - 0,9123	1,70 (20,67%)	0,8737	0,7942 - 0,9182	1,70 (20,24%)
Eroded area (cm2)	0,8427	0,8108 - 0,8691	1,03 (69,35%)	0,6331	0,4593 - 0,7466	1,26 (84,51%)
Pico index	0,8619	0,8338 - 0,8852	0,08 (45,78%)	0,7560	0,6148 - 0,8390	0,10 (56,38%)
Gerber's index	0,7697	0,7244 - 0,8075	0,21 (20,66%)	0,6039	0,4866 - 0,6982	0,26 (25,65%)

### Inter-observer reliability

Superior-inferior height and anterior-posterior width showed excellent ICC results with excellent MDC95 (Table 2). Radius, Circle Area presented good ICC and good MDC95% values. Pico Index had a good ICC value but not acceptable MDC95%. Erosion edge and Eroded area presented moderate ICC values with not acceptable MDC95%. Gerber’s index showed moderate ICC and good MDC95%.

### Impact of learning

When compared the first 30 measured scans vs the last 30 results clearly showed that ICC and MDC95% improved in all parameters (Table 3) for particular evaluator (intra-observer reliability).

**Table 3:** Results of impact of learning on intra-observer reliability, comparing first 30 patients (A) vs last 30 patients (B). (ICC- intraclass correlation coefficient, MDC95 - minimal detectable change with 95% confidence)

Parameter	ICC A	ICC B	95% CI A	95% CI B	MDC95 (%) A	MDC95 (%) B
Superior-inferior height (cm)	0,8611	0,9363	0,8138 - 0,8964	0,9145 - 0,9525	0,43 (10,09%)	0,31 (7,18%)
Anterior-posterior width (cm)	0,9150	0,9156	0,8860 - 0,9366	0,8862 - 0,9373	0,28 (10,71%)	0,29 (10,47%)
Erosion edge (cm)	0,7105	0,8936	0,6119 - 0,7841	0,8573 - 0,9206	1,06 (32,58%)	0,66 (19,70%)
Radius (cm)	0,8604	0,9476	0,8095 - 0,8972	0,9296 - 0,9610	0,39 (12,41%)	0,24 (7,33%)
Circle area (cm2)	0,8644	0,9404	0,8154 - 0,9000	0,9199 - 0,9556	1,91 (23,80%)	1,31 (15,23%)
Eroded area (cm2)	0,7919	0,8909	0,7193 - 0,8455	0,8537 - 0,9186	1,10 (83,34%)	0,81 (56,74%)
Pico index	0,8066	0,9088	0,7397 - 0,8561	0,8776 - 0,9320	0,10 (60,95%)	0,07 (41,85%)
Gerber's index	0,6668	0,8395	0,5531 - 0,7516	0,7848 - 0,8803	0,27 (26,67%)	0,19 (18,51%)

*n*=180

When compared Inter-observer reliability of the first 30 measured patients vs the last 30 (Table 4), MDC95% and ICC improved in some parameters (Superior-inferior height, Circle area) but remained mostly unchanged or even worsened in the remaining ones (Anterior-posterior width, Erosion edge, Radius, Eroded area, Pico index, Gerber’s index).

**Table 4:** Inter-observer reliability change - first 30 patients (A) vs last 30 patients (B) (ICC- intraclass correlation coefficient, MDC95 - minimal detectable change with 95% confidence)

Parameter	ICC A	ICC B	95% CI A	95% CI B	MDC95 (%) A	MDC95 (%) B
Superior-inferior height (cm)	0,8923	0,9286	0,8359 - 0,9318	0,8529 - 0,9620	0,36 (8,48%)	0,30 (7,08%)
Anterior-posterior width (cm)	0,9669	0,9617	0,9520 - 0,9783	0,9445 - 0,9750	0,18 (6,69%)	0,20 (7,05%)
Erosion edge (cm)	0,7346	0,4738	0,6148 - 0,8263	0,1914 - 0,6704	0,97 (29,88%)	1,03 (30,61%)
Radius (cm)	0,9089	0,8944	0,8626 - 0,9420	0,7440 - 0,9485	0,31 (9,73%)	0,30 (9,03%)
Circle area (cm2)	0,8988	0,8826	0,8464 - 0,9358	0,7249 - 0,9418	1,59 (19,79%)	1,60 (18,51%)
Eroded area (cm2)	0,7390	0,4246	0,6130 - 0,8316	0,1474 - 0,6300	1,06 (80,46%)	1,14 (79,96%)
Pico index	0,8647	0,5914	0,7983 - 0,9131	0,2729 - 0,7701	0,07 (44,61%)	0,09 (55,37%)
Gerber's index	0,7397	0,3937	0,6239 - 0,8292	0,1534 - 0,5890	0,23 (22,18%)	0,28 (27,78%)

*n*=60

## Discussion

**Fig. 3:**
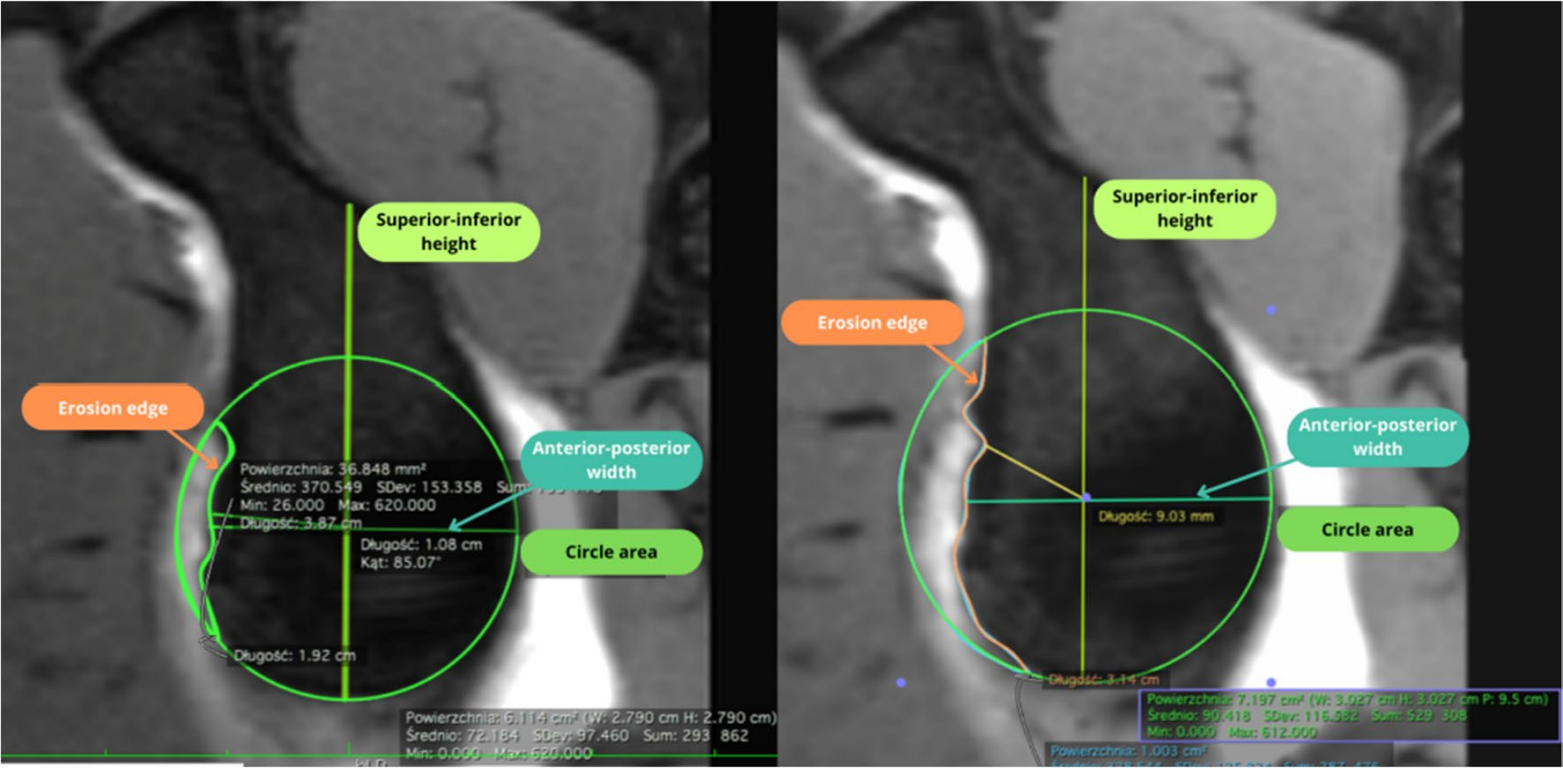
Example of difference in measurements between two investigators on the same MRI scan

In this study we presented that MPR MRI measurement of the anterior glenoid lesions is valuable for some measurement and but has some limitation for more complex assessment. It has very good reliability for linear parameters, but does not seem very good for assessing the glenoid defect area (and therefore Pico index). MRI imaging, due to its obvious benefits and improved quality, is becoming a valuable alternative to CT imaging in orthopedics even for bone evaluation. Some studies show that both may have comparable quality for assessing the glenoid bone loss, thus making MRI a radiation-free alternative to preoperative shoulder scans [6, 8, 19]. Yet some still have concerns regarding this method [20] (Fig. 3).

Generally speaking, imaging techniques reflect reality better than 2D. Studies on glenoid measurements using CT showed that 3D imaging has excellent reliability for all studied glenoid parameters, while 2D had acceptable results only for some of them [7]. 3D imaging appears to facilitate proper glenoid plane alignment and more accurate measurements. Results for MRI are less conclusive. We couldn’t find any studies directly comparing 3D and 2D MRI for glenoid assessment. Some conclusions can be drown from the studies comparing MRI and CT. A study comparing 2D MRI and 2D CT showed that both techniques have almost similar results for glenoid width, anterior straight line length and glenoid bone loss [21]. Comparison of 3DMR and 3DCT measurements showed that there is no significant difference between the two techniques for some glenoid parameters - surface arena, height and width (although very small number of images were studied, only 7, with artificially created bone loss - cadaveric study) [22]. The MPR MRI used in our study, although not a full 3D reconstruction, allows proper positioning of the glenoid. Measurements using this technique do not require special software and time-consuming reconstruction

Our results suggest that linear measurements, such as superior-inferior height or anterior-posterior width, are characterized by very high reliability. They are easy to learn, even for inexperienced researchers, to measure accurately. The problem arises with more complex, area measurements such as the Circle area or Eroded area, which directly influence the Pico index. Although the technique for performing these measurements seems simple, significant differences between researchers arise. There seem to be a lot of room for arbitrary and subjective assessment of best fit circle based on not very sharp contrasts on MR scan. This problem was observed in previous studies, which showed that additional steps (such as extending a line perpendicular to the midpoint of the supraglenoid tubercle across the glenoid) can be added to improve the reliability of Pico measurements [23]. Moreover, our study suggests that the accuracy of glenoid measurement depends on experience with this technique. This was also observed in a previous study, where authors stated that the were reliability differences between more and less experienced radiologists [24].

When we compare our results with available literature, partially similar results were obtained in a study by Gyftopoulos et al., which measured glenoid loss using standard 2D MRI. Although limited in sample size (only 18 cadaveric glenoids), the authors showed that for the circle method (Pico), inter-observer reliability is only moderate while intra-observer reliability is excellent. Other parameters were not described in the study [23]. Interestingly, the authors also underline the effect of learning on the accuracy of measurement. This corresponds with our results, which clearly show that experience plays a crucial role in glenoid measurement. Interesting study by Sgroi et al. showed that 2D MRI, for both linear and surface area parameters have very good inter-rater and intra-rater reliability, with no significant difference to CT. The differences between our results may be due to the different approach and technical differences. Study by Sgroi et al. used standard 2D MRI while we used MPR reconstruction. Also observers in cited study started real measurements only when the respective intrarater reliabilities were good. In our study we did not assess the reliability of the researchers' measurement before the actual measurements were taken. Another study by Gyftopoulos et al. discussed the usefulness of 3D MRI for the evaluation of glenoid bone loss. The authors used the 3D-T1W-FLAShaveH sequence, and two observers measured images of 15 patients. The results showed that ICC for PICO inter-observer reliability was very good, which is a better result than what was obtained in our study. The difference might arise from the fact that the authors of the study used "real" 3D reconstruction, while in our study we used MPR MRI, which is a less advanced technique. For other parameters than PICO, we found a study by de Mello et al. which showed excellent inter and intra-rater reliability for glenoid width using 3D MRI. Although the study group comprised only 10 patients, these findings are consistent with our results [25].

Our study seems to be the first one with a relatively large study group (80 patients) and six observers. Most of the studies that we found were based on 20 images or fewer, which should not be overlooked when drawing conclusions. Based on our results, we suggest that MPR MRI should be used cautiously when it comes to glenoid defect measurement using the PICO method. "Real" 3D MRI is a more complex but definitely a better alternative.

MPR MRI seems to be a compromise between 2D and 3D MR imaging. On the one hand, it does not have all the benefits of 3-dimensional imaging, but on the other hand, it is much simpler to produce. There are important differences between MRI and CT techniques that potentially affect the reliability of glenoid measurements. CT images often have higher spatial resolution than MRI images for bone structures. An additional difficulty is the inferior differentiation between bone and soft tissue on MRI. This difference affects measurement reliability and makes MRI more difficult to masters. We think that it might be especially important for young clinicians, who do not have vast experience in glenoid measurements. The reliability of measurement is not independent of the researcher's experience. Although it seems to be less important for 3D imaging [8]. Therefore, we believe that especially young clinicians should have access to 3D techniques that are easier to assess. This conclusion is supported by other researchers [7, 26]. It seems that in the future, limitations in the reliability of glenoid measurements might be solved by machine learning models [27]. Advancements in technology could mitigate the human factor, which appears to be a significant limitation.

Our study has limitations. We have no direct comparison to the gold standard of 3D CT. Such comparison would allow for deeper validation yet would include exposing patients for radiation and at the stage was avoided for the project. The observers involved in the study were not experienced doctors but medical students who underwent previous training on glenoid measurement. Yet on the other hand that allows us to really assess the value of measurement that could be questioned if done be not experienced evaluator. Secondly, the observers were focused on finding glenoid defects, which sometimes might not be present in the images. This could potentially lead to confirmation bias. Yet this was based on set of consecutive instability patients, that represents clinical scenario. In our study we used high resolution, 3 Tesla MRI, with Volumetric interpolated breath-hold examination (VIBE). This is possible only on certain MRI machines. Another limitations of the study and technique of measurements is time of whole process. The procedure is time consuming. That clearly is dependent on the experience of the evaluator. We have not measured the average time of the measurements and that seemed rather obvious. We also believe that in the future machine learning and artificial intelligence will facilitate the whole process. So can be probably achieved with 3D reconstructions based on MR, as we have proven 3D measurement superiority over 2D one.

## Conclusions

To conclude, high resolution MPR MRI measurement of the anterior glenoid lesion is very good tool for linear glenoid parameters. It is simple to implement and resistant to the human factor and low experience. On the other hand, however, clinically more important area measurements, such as Pico index measurement or the area of bone loss is unreliable using MPR MRI. Attention should also be drawn to the pace of learning, which is very individual. Complex, area measurements based on MPR MRI are not resistant to low experience.
